# Sequencing and *De Novo* Analysis of the Hemocytes Transcriptome in *Litopenaeus vannamei* Response to White Spot Syndrome Virus Infection

**DOI:** 10.1371/journal.pone.0076718

**Published:** 2013-10-18

**Authors:** Shuxia Xue, Yichen Liu, Yichen Zhang, Yan Sun, Xuyun Geng, Jinsheng Sun

**Affiliations:** 1 Tianjin Key Laboratory of Animal and Plant Resistance, College of Life Science, Tianjin Normal University, Tianjin, People’s Republic of China; 2 Tianjin Center for Control and Prevention of Aquatic Animal Infectious Disease, Tianjin, People’s Republic of China; Chang Gung University, Taiwan

## Abstract

**Background:**

White spot syndrome virus (WSSV) is a causative pathogen found in most shrimp farming areas of the world and causes large economic losses to the shrimp aquaculture. The mechanism underlying the molecular pathogenesis of the highly virulent WSSV remains unknown. To better understand the virus-host interactions at the molecular level, the transcriptome profiles in hemocytes of unchallenged and WSSV-challenged shrimp (*Litopenaeus vannamei*) were compared using a short-read deep sequencing method (Illumina).

**Results:**

RNA-seq analysis generated more than 25.81 million clean pair end (PE) reads, which were assembled into 52,073 unigenes (mean size = 520 bp). Based on sequence similarity searches, 23,568 (45.3%) genes were identified, among which 6,562 and 7,822 unigenes were assigned to gene ontology (GO) categories and clusters of orthologous groups (COG), respectively. Searches in the Kyoto Encyclopedia of Genes and Genomes Pathway database (KEGG) mapped 14,941 (63.4%) unigenes to 240 KEGG pathways. Among all the annotated unigenes, 1,179 were associated with immune-related genes. Digital gene expression (DGE) analysis revealed that the host transcriptome profile was slightly changed in the early infection (5 hours post injection) of the virus, while large transcriptional differences were identified in the late infection (48 hpi) of WSSV. The differentially expressed genes mainly involved in pattern recognition genes and some immune response factors. The results indicated that antiviral immune mechanisms were probably involved in the recognition of pathogen-associated molecular patterns.

**Conclusions:**

This study provided a global survey of host gene activities against virus infection in a non-model organism, pacific white shrimp. Results can contribute to the in-depth study of candidate genes in white shrimp, and help to improve the current understanding of host-pathogen interactions.

## Introduction

Pacific white shrimp (*L. vannamei*) is one of the locally cultured shrimp in China and plays a major role in the Chinese mariculture industry. However, outbreak of disease caused by WSSV becomes the major stumbling blocks in the development and sustainability of shrimp aquaculture. WSSV is a large dsDNA virus infecting crustaceans and is the most important viral pathogen of cultured shrimp worldwide [1]–[4]. In cultured shrimp, WSSV causes a cumulative mortality of up to 100% within 3–10 days [5]. Because of its rapid spread and high associated mortality rates, WSSV is an extremely virulent pathogen in shrimp culture.

Researchers so far have concentrated mainly on individual genes or small defined groups of host or WSSV genes, to explore their function or differential expression. However, there is still no effective treatment available to interfere with the unrestrained occurrence and the spread of the disease. An understanding on the interaction between host and pathogen will be helpful in controlling the infectious diseases in shrimp.

Deep sequencing data can provide extensive information about host-virus interactions at the transcriptional level. More recently, direct sequencing of transcripts by high-throughput sequencing technologies (RNA-seq) has become an additional alternative to microarrays and is superseding SAGE and MPSS. Transcript profiling offers the largest coverage and a wide dynamic range of gene expression information and can often be performed genome wide. The large amounts of sequencing data that can readily be produced by next-generation sequencing platforms, such as Solexa/Illumina RNA-seq and Digital gene expression (DGE), reduces the need for prior sequence knowledge on gene expression profiling and are now making direct sequencing approaches the method of choice for whole transcriptome analysis in many species [6]–[15].

RNA-seq refers to whole transcriptome shotgun sequencing wherein mRNA or cDNA is mechanically fragmented, resulting in overlapping short fragments that cover the entire transcriptome. DGE is a tag-based transcriptome sequencing approach where short raw tags are generated by endonuclease. The expression level of all genes in the sample is measured by counting the number of individual mRNA molecules produced from each gene. These two technologies have been used in transcriptome profiling studies for various applications.

In the present study, a deep sequencing approach was used to give a comprehensive view of immune-related genes that are differentially expressed in the early and late WSSV infection stage, revealing a significant number of genes which have not been reported previously. This study might provide new clues on WSSV control by identifying molecules that are important for immune defense on *L. vannamei*, viral resistance and other important metabolic regulatory functions.

## Materials and Methods

### Shrimp, Virus and Challenge

Hundred healthy shrimp (*L. vannamei*), 15.2 g in average body weight, were obtained from a shrimp farm in Hainan province of China and reared in fiberglass tanks. The shrimps were fed with clam meat for 7 days and acclimatized to the laboratory conditions which the salinity (25‰) and temperature (21°C) were maintained at the same levels as that of the shrimp culture ponds. WSSV-free shrimps were detected by PCR amplification [16]. For the WSSV challenge experiment, each experimental and control animal were injected with 10 µL (5.96×10^6^ copies/ml, identified by qPCR) WSSV suspension prepared from WSSV-infected and mock PBS, respectively.

### Sample Preparation

Approximately 300 µl hemolymph was obtained from each shrimp. Hemolymph of 8–10 shrimps in each group were collected and pooled at 5 h and 48 h post WSSV-challenge. Total RNAs of control and WSSV-challenged shrimp were obtained from hemocytes by using TRIzol reagent (Gibco BRL) following the manufacturer’s instructions and treated with RNase free DNase I (Qiagen). RNA integrity was confirmed by 2100 Bioanalyzer (Agilent Technologies). The samples used had a minimum RIN value of 7.0.

For RNA library construction and deep sequencing, equal quantities of RNA from each group were pooled. Approximately 10 µg of RNA representing each group were submitted to Illumina HiSeq™ 2000 for sequencing. In brief, mRNAs were purified using oligo-dT-attached magnetic beads and fragmented into small pieces (200 nt–700 nt) in a fragmentation buffer. Cleaved RNA fragments were copied into first strand cDNA using reverse transcriptase and random hexamers. This was followed by second strand cDNA synthesis using DNA polymerase Ι and RNase H. These cDNA fragments underwent end repair process and ligation of adapters. Products were subsequently purified and amplified through PCR to create the final cDNA libraries.

### Analysis of Illumina Sequencing Results

The cDNA library was sequenced on the Illumina HiSeq^TM^2000 sequencing platform. Both ends of the library were sequenced. The 90-bp raw PE (paired end) reads were cleaned by removing adaptor sequences, empty reads and low quality sequences (reads with unknown sequences ‘N’). The clean reads were submitted to EBI European Nucleotide Archive (ENA) database (http://www.ebi.ac.uk/ena/data/view/PRJEB4317, accession number: PRJEB4317). Short reads assembly software Trinity [17] was used for *de novo* transcriptome assembly. Firstly, overlaps generated from assembling of clean reads were connected into longer fragments called contigs. Then, all PE reads were compared back with contigs. Overlap of PE reads with two contigs was taken to indicate that the contigs were short segments of a scaffold. Reads were used for gap-filling of these scaffolds to generate final scaffold sequences. Finally, the scaffolds were clustered using TGI Clustering tools, and distinct gene sequences called unigenes were generated. Unigenes with a minimum length of 200 bp were selected and submitted to EBI European Nucleotide Archive (ENA) database (http://www.ebi.ac.uk/ena/, accession number: HAAW1000001–HAAW01042151). Sequence homology searches were performed using local BLASTall programs against sequences in NCBI non-redundant (nr/nt) protein/nucleotide database and the Swissprot database (E-value <1e-5) [18]. Functional annotation by gene ontology terms (GO; http://www.geneontology.org) was analyzed by Blast2go and WEGO software [19]–[20]. The COG and KEGG pathways annotation was performed using Blastall software against Cluster of Orthologous Groups database and Kyoto Encyclopedia of Genes and Genomes database. To predict coding sequences (CDS), unigenes were compared with nr, Swiss-Prot, KEGG and COG database according to priority order. If a unigene was annotated in a high priority database, it would not compare with other databases any more. Otherwise, the unigene would compare with the next database automatically. We took the highest rank proteins to predict the CDS of the unigenes, and then the coding sequences were translated into amino acid sequences based on the standard anticodon table. The CDS of unigenes which cannot be compared with annotated in the four databases were predicted using ESTscan software [21].

### Digital Gene Expression Library Preparation and Sequencing

Tag library preparation for the three shrimp groups was performed in parallel using Illumina gene expression sample preparation kit. Briefly, 2 µg total RNA of each group was used for mRNA capture with magnetic oligo-dT beads. First and second strand cDNA were synthesized and bead-bound cDNA was subsequently digested with Nla III, which recognizes and cuts off the CATG sites on cDNA. Fragments other than the 3′ cDNA fragments attached to oligo-dT beads were washed away and Illumina adapter 1 was added to the 5′-ends. The adapter 1 contains a restriction site for Mme I which cuts 17 bp downstream from the CATG site, thereby releasing 21 bp tags. A second adapter was ligated at the site of Mme I cleavage, and the adapter-ligated cDNA tags were enriched after 15 cycles of linear PCR amplification. The resulting 85-bp fragments were purified from a 6% acrylamide gel. Fragments were then digested and the single-chain molecules were fixed onto the Illumina Sequencing Chip (flowcell). Sequencing by synthesis was performed using the Illumina HiSeq™ 2000 system according to the manufacturer’s protocols. Image analysis, base calling, generation of raw 17-bp tags, and tag counting were performed using the Illumina pipeline.

### Mapping of DGE Tags

Prior to mapping reads to the reference database, we filtered all reads to remove adaptor read, low quality reads (percentage of unknown base ‘N’ was more than 10%), empty tags (sequence with only adaptor sequence but no tags). All clean tags were mapped to the reference transcriptome generated by RNA-seq using SOAPaligner/soap2 software [22]. For annotation, all tags were mapped to the reference sequences and only allowed no more than 2 nucleotide mismatch. For gene expression analysis, the number of expressed tags was calculated and then normalized to TPM (number of transcripts per million tags); and the differentially expressed tags were used for mapping.

### Identification of Differentially Expressed Genes

Statistical comparison was performed using RPKM (Reads Per Kb per Million reads) method which was described by Mortazavi *et al* [23]. In addition to P value, FDR (false discovery rate) was used to determine differentially expressed genes in multiple test and analysis [24]. In this research, we used P≤0.01, FDR≤0.001 and the absolute value of log2Ratio≥1 as the threshold to judge the significance of gene expression difference. For pathway enrichment analysis, we mapped all differentially expressed genes to terms in KEGG database and looked for significantly enriched KEGG terms compared to the genome background.

### Experimental Validation

Representative unigenes with complete ORFs including chymotrypsin BI (unigene18988), ubiquitin-conjugating enzyme E2 (unigene8925), argonaute 2 (unigene7508) and HSP70 (unigene1876) generated by RNA-seq were selected for cloning and sequencing validation. All PCR products were purified using a QIAqucik PCR Purification Kit (Qiagen), cloned into the pMD-18T vector (TaKaRa) and transformed into TOP10 competent cells. The positive clones were sequenced in Sangon Biotech (Shanghai) Co., Ltd. Furthermore, four genes (penaeidin4a, peroxinectin, glucan-binding protein and peroxiredoxin) were selected for qPCR confirmation. The RNA samples used for the qPCR assays were both the same as for the DGE experiments. qPCR were done on the Lightcycler iQ^TM^5 (Roche), with SYBR PrimeScript RT-PCR Kit (TaKaRa Biotechnology Co., Ltd.), according to the manufacture’s instruction. Melting curves for each sample were analyzed to check the specificity of amplification. Each sample was analyzed in triplicate and the average threshold cycle (Ct) was calculated with the 2^−△△Ct^ method. The results were normalized to the expression level of triosephosphate isomerase (TPI) and relative to the control group.

## Results

### Sequencing and *de novo* Assembly of the Transcriptome

**Figure 1:**
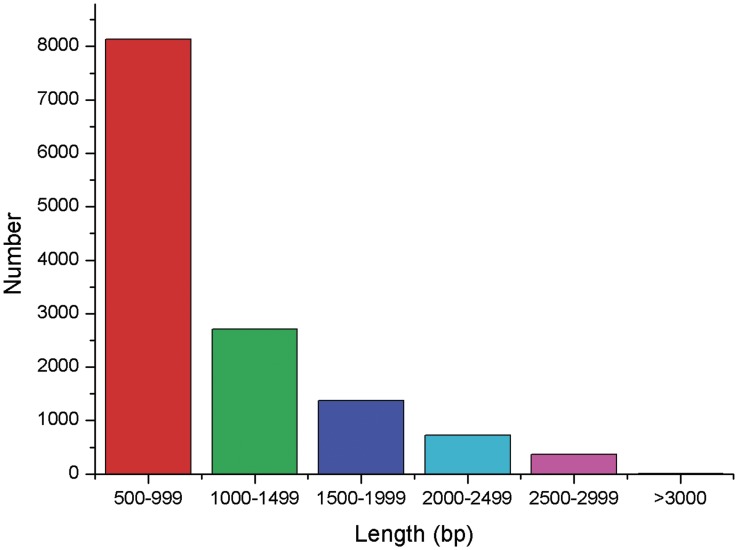
Distribution of unigenes size (>500 bp) in assembled *L. vannamei* transcriptome.

A transcriptome is the complete set of expressed RNA transcripts in one or more cells. Transcriptome profiling of *L. vannamei* under WSSV challenge helps us to obtain a better understanding of subsequent related cellular activities in organisms including growth, development, and immune defense. To obtain an overview of the *L. vannamei* gene expression profile, a cDNA library was prepared from the three groups mentioned above and sequenced using Illumina HiSeq^TM^2000 system. Approximately 28.24 million 90-bp pair end (PE) raw reads were generated from one plate (8 lanes) of sequencing. Afterwards, repetitive, low complexity, and low quality reads were filtered out and about 25.81 million clean reads were obtained for non-redundant consensus. Then, the short clean reads were assembled using Trinity *de novo* assembly software and presented 101,479 contigs. The mean contig size was 296 bp with lengths ranging from 100 bp to over 3000 bp (median length of all non-redundant consensus sequences, N50 = 430 bp). Using paired-end joining and gap-filling, the contigs were further assembled into scaffold sequences. Using trinity software programs, the scaffold sequences were clustered and generated 52,073 consensus unigenes (http://59.67.75.245/college/skxy/skin/one/show1.asp?id=2044) with 728 distinct clusters and 51,345 distinct singletons (Table 1). The mean unigene size was 520 bp with lengths ranging from 100 bp to over 3000 bp (N50 = 745 bp). The distribution of unigenes size is shown in Figures 1. These sequences provide abundant data for better understanding the hemocytes transcriptome of *L. vannamei* infected with WSSV.

**Table 1:** Summary of the transcriptome.

Total raw reads	28,243,652
Total clean reads	25,814,054
Total number of contigs	101,479
Mean length of contigs	296 bp
Total unigenes	52,073
Mean lengte of unigenes	520
Distinct clusters	728
Distinct singletons	51,345

### Annotation of Non-redundant Unigenes

**Figure 2:**
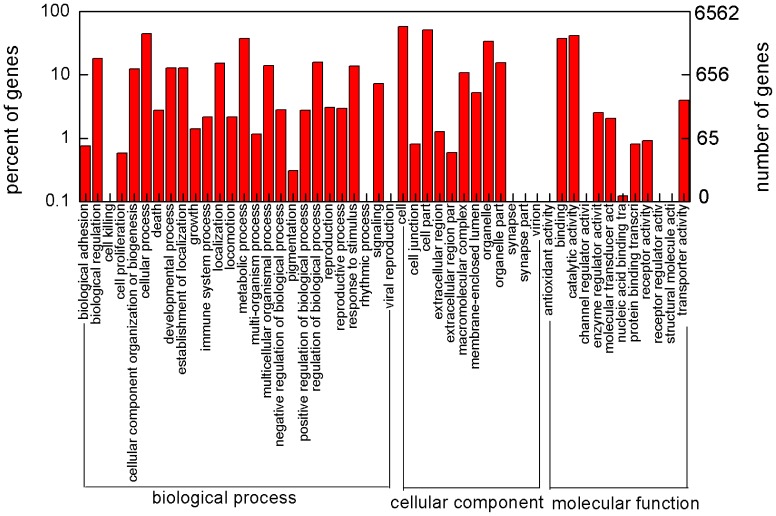
GO annotations of consensus sequences. Best hits were aligned to the GO database, and 6,562 consensus sequences were assigned to at least one GO term. This program categorized 14,872 unigenes in biological process, 11,585 unigenes in cellular component and 5,837 unigenes in molecular function.

**Figure 3:**
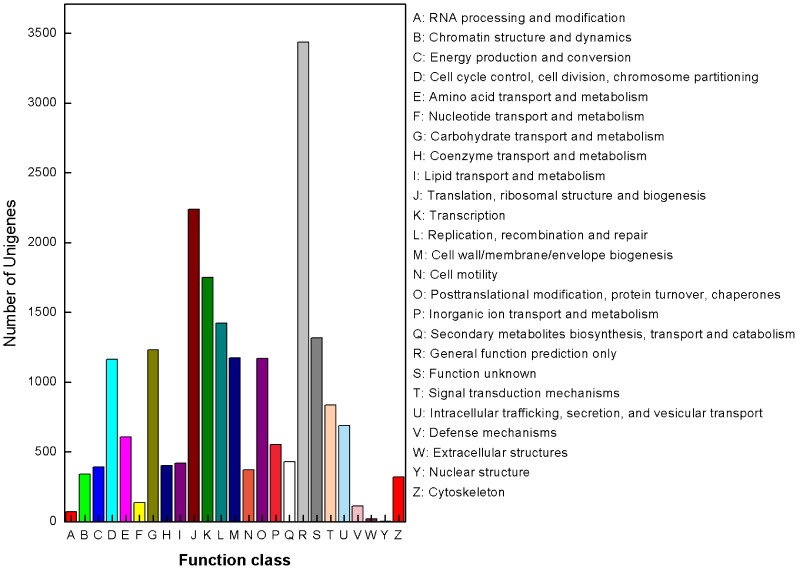
COG annotations of putative proteins. All putative proteins were aligned to the COG database and can be classified functionally into at least 25 molecular families.

**Figure 4:**
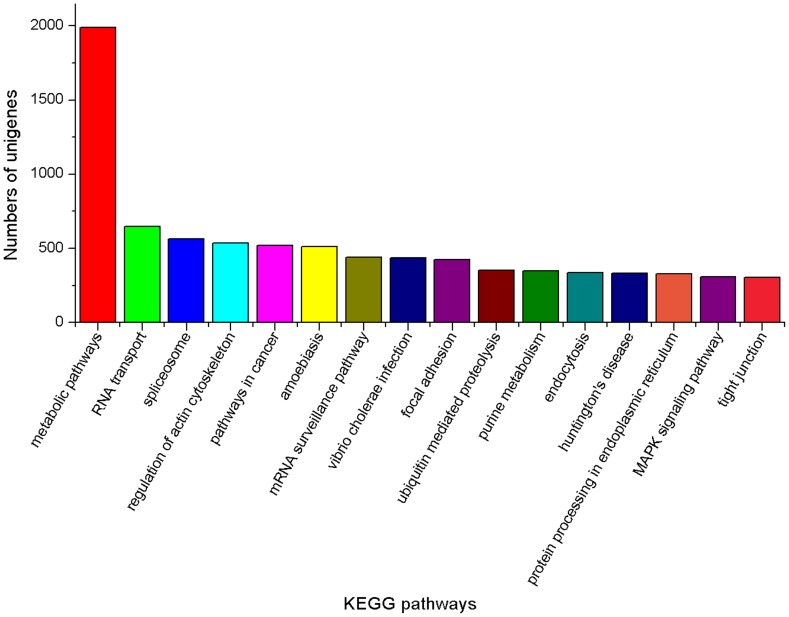
KEGG classifications of the unigenes. 14,941 unigenes were assigned into 240 pathways. The pathways which were mapped by more than 300 unigenes were shown.

For annotation, 52,073 unigenes were analyzed using local BLASTall programs against nr/nt and SwissProt database with a cut-off E-value of 10^−5^. Using this approach, 23,568 unigenes (45.3% of all distinct sequences) returned an above cut-off BLAST result (Table 2). Comparison with the Nr database showed that 20,343 unigenes had high similarity with known gene sequences in existing species. Then, Blast2GO and WEGO software [19], [25] were used to annotate the 20,343 unigenes by searching against GO database. Blastall software was used to search COG database. Good results of 6,562 consensus sequences and 7,822 putative proteins [6], [7] were yielded, respectively. GO assignments were used to classify the functions of the predicted unigenes. All the annotated unigenes belonged to the biological process, cellular component, and molecular function clusters and distributed among 50 categories, including metabolism, growth, development, apoptosis, biochemistry, immune defense, molecular processing, signal transduction, transcription regulator activity etc (Figure 2). Similarly, COG-annotated putative proteins were classified functionally into at least 25 molecular families, including RNA processing and modification, energy production and conversion, nucleotide transport and metabolism, nuclear structure, defense mechanisms, signal transduction mechanisms, cytoskeleton etc (Figure 3). The KEGG database was used to analyze potential involvement of unigenes in cellular metabolic pathways. Among the 23,568 unigenes, 14,941 can be grouped into 240 known pathways, including various signaling or metabolic pathways, apoptosis, differentiation, and cellular growth (Figure 4).

**Table 2:** Annotation of unigenes.

Database	Number of annotatedunigenes	Percentage of annotatedunigenes
Nr	20,343	86.3%
Nt	10,341	43.9%
SwissProt	17,677	75.0%
KEGG	14,941	63.4%
COG	7,822	33.2%
GO	6,562	27.8%

### Annotation of Immune-related Genes and Pathways

To obtain deep insight into the molecular biology of innate immune systems in *L. vannamei*, the immune-related genes were analyzed. Approximately 1,179 unigenes were found to be homologous with known immune-related genes in other species (Table 3, Table S1). KEGG analysis revealed that 963 immune-related unigenes were significantly enriched in various known metabolic or signaling pathways, such as Toll-like receptor signaling pathway, ubiquitin mediated proteolysis, MAPK signaling pathway, jak-STAT signaling pathway, and calcium signaling pathway etc.

**Table 3:** Immune-related genes/homologues in *L. vannmei.*

Putative Gene Catalogs	Unigenes Number		Putative GeneCatalogs	UnigenesNumber	
*Pattern recognition genes*			Rab		34
C-type lectin		52	Rac		6
TLR and revelent		15	Rho		42
mannose receptor		26	NFγ		4
peptidoglycan-bindingprotein		6	STAT		6
glucan-binding protein		9	*Other Immune* *Response Factors*		
LPS anchor protein		1	hemocyanin		20
LRR		83	α-macroglobulin		26
scavenger receptor		10	ALF		7
*Phenoloxidase System*			PEN		6
phenoloxidase and revelent		28	HSP		36
serine protease and revelent		100	argonaute		12
*Antioxidant Enzymes*			dicer		19
superoxide dismutase		6	lysozyme		5
catalase		2	chitinase		26
peroxidase		19	agmatinase		3
NADPH oxidase		8	thrombospondin		3
peroxiredoxin		5	oxygenase		13
*Programmed Cell Death Protein*			PCNA		1
caspase		26	peroxinectin		5
apoptosis revelent		107	ferritin		6
*Ubiquitin Proteasome* *Pathway Revelent*		187	selenoprotein		8
*cell adhension molecules*			calpain		4
cadherin		23	arginine kinase		1
integrin		44	calreticulin		1
*Signal Transduction* *Molecule*			crustin		9
MAPK		12	kazal-typeproteinaseinhibitor		11
JNK		2	relish		6
ERK		1	spatzle protein		1
Myd88		1	thioredoxinperoxidase		3
Ras		82			

Putative genes were confirmed based on their similarity to immune-related genes in other invertebrate species as predicted by BLAST. Cutoff E-value was set to 1e-5 during BLAST analysis. Characterization of the putative genes is presented in Additional file 1.

### Digital Gene Expression Library Sequencing

Illumina DGE can generate absolute rather than relative gene expression measurements and avoids many of the inherent limitations of other methods, such as microarray analysis. In the present study, we sequenced three DGE libraries: mock sample, early infection sample (5hpi) and late infection sample (48hpi), and generated 6.28, 6.05 and 6.03 million raw tags from each of the three samples, respectively. After removing adaptors and low quality reads, a total number of 6.25, 6.02 and 5.98 million high qualities non-redundant tags of the mock, early infection and late infection samples were identified by base calling [26]–[27].

### Assessment of the DGE Libraries

**Figure 5:**
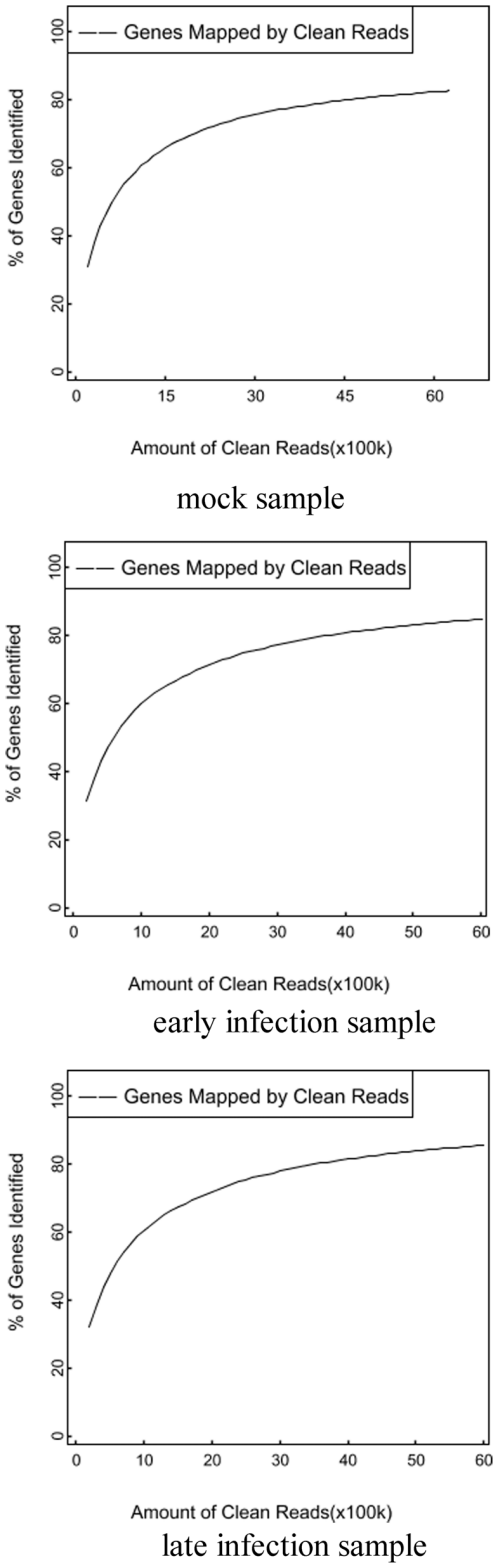
Saturation analyses of DGE clean reads generated from mock, early infection and late infection samples. With the number of reads increasing, the number of detected genes is increasing. When the number of reads reaches certain value, the growth rate of detected genes flattens, and it means that the number of detected genes tends to saturation.

To confirm the number of detected genes increases proportionally to sequencing amount, a saturation analysis was performed. Sequence saturation analysis is used to measure the sequencing data of a sample. With the number of reads increasing, the number of detected genes is increasing. However, when the number of reads reaches certain value, the growth rate of detected genes flattens, and it means that the number of detected genes tends to saturation. Figure 5 shows a trend of saturation where the number of detected genes almost ceases to increase when the number of reads reaches about 6 million.

**Figure 6:**
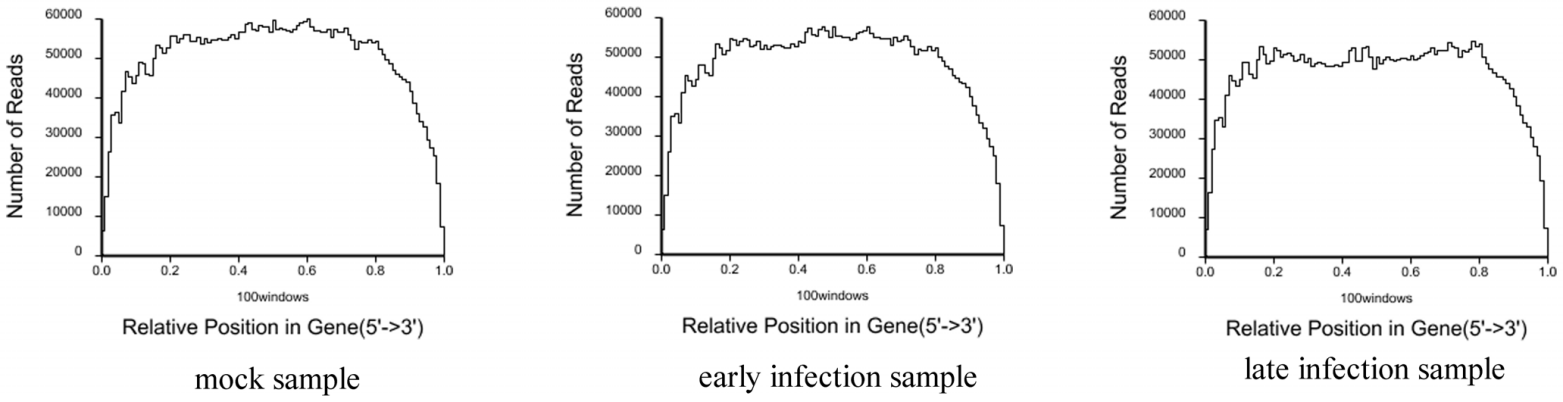
Distribution statistics of reads mapped to reference genes. The horizontal axis represents the relative reads position which is calculated as the ratio between reads location on the gene and gene length; the vertical axis represents the number of reads that mapped to genes.

During preparing the DGE sequencing libraries, mRNA were firstly broken into short segments by chemical methods and then sequenced. If the randomness of breaking is poor, reads preference from specific gene region will affect the subsequent analysis directly. We used the distribution of reads locating on the genes to evaluate the randomness of breaking. Since genes have different lengths, the reads location on gene is standardized to a relative position (which is calculated as the ratio between reads location on the gene and gene length), and then the number of reads in each position is counted [28]. From the evenly distributed reads in every position of genes, it could be concluded that the randomness of breaking of the three samples is good (Figure 6).

### Mapping Tags to the Reference Transcriptome

**Figure 7:**
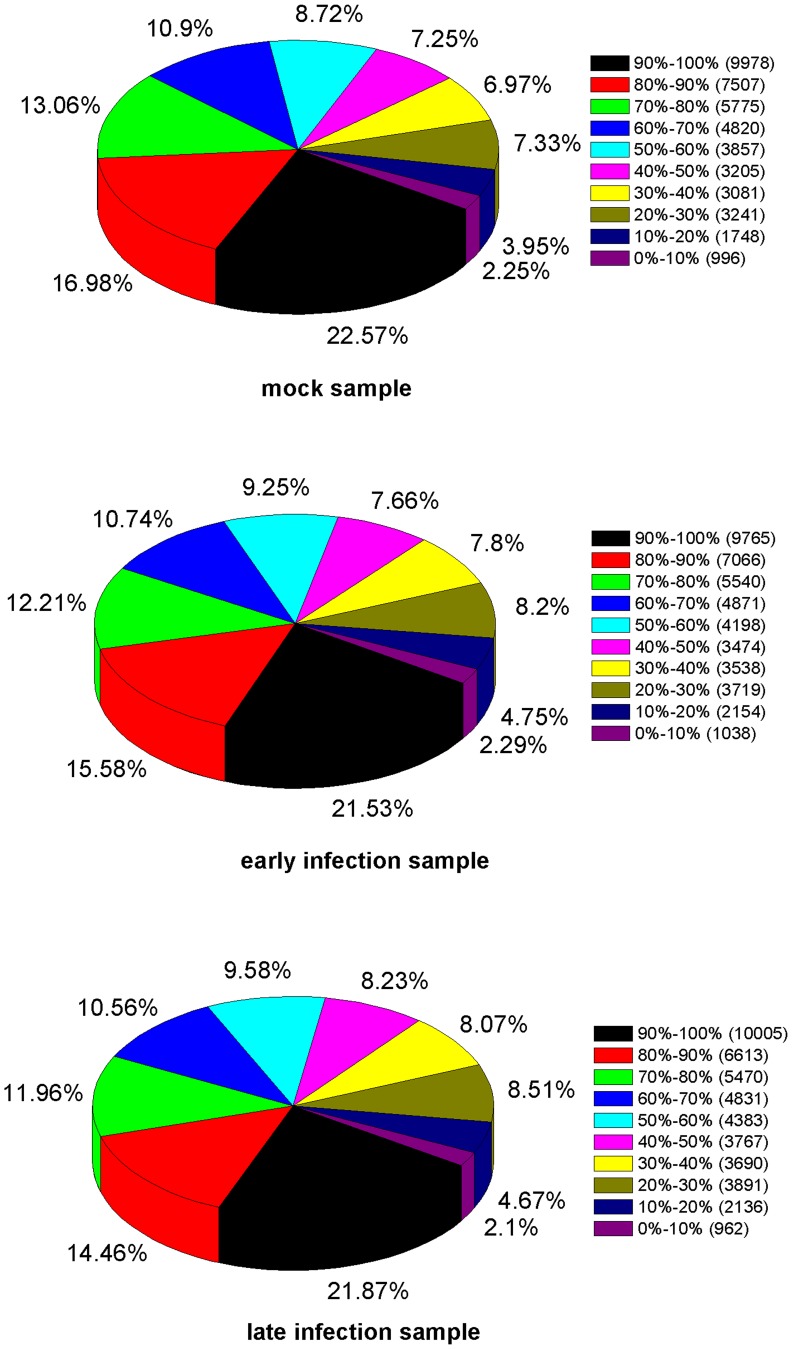
Distributions of genes’ coverage of mock, early infection and late infection samples. Genes’ coverage is used to assess the depth of DGE sequencing. It refers to the percentage mapped by reads of each gene.

Tag mapping analysis showed that 79.37%, 79.79% and 77.06% of all distinct tags in the three groups could be mapped to the reference database provided by the 52,073 non-redundant unigenes from the RNA sequence based transcriptome analysis. Tags mapped to a unique sequence are pivotal subset of the DGE libraries as they can identify a transcript explicitly. The gene expression is calculated by the numbers of reads mapped to the reference sequence and every gene. Figure 7 shows the distribution of genes’ coverage of the three samples.

### Changes in Gene Expression Profile After WSSV Challenge

To analyze the global transcriptional changes in shrimp at the early and late virus infectious stage, the method described by Audic *et al*. [29] was applied to identify differentially expressed genes from the normalized DGE data by comparisons between the mock- and WSSV-challenged samples. Between the mock and early infection samples, only 315 differentially expressed genes were detected with 227 up-regulated genes and 88 down regulated genes (Table S2). However, 4,226 differentially expressed genes with 2,506 up-regulated genes and 1,720 down-regulated genes were identified between the mock and late infection samples (Table S3). Among the differentially expressed genes identified in the late infection samples, a total of 1,672 genes were annotated in the NCBI database, including 527 hypothetical proteins, 34 predicted proteins and 12 unnamed proteins. From the remaining 1,099 annotated genes, we screened 190 genes which were confirmed as immune related genes in the previous study. All these screened genes contained pattern recognition receptors and some immune factors (Table S4).

### Functional Annotation of Differentially Expressed Genes

To understand the function of differentially expressed genes, all genes in this research were mapped to terms in GO and KEGG database. 24 (cellular component ontology), 46 (molecular function ontology) and 44 (biological process ontology) differentially expressed genes were identified between the mock and early infection samples; while 463 (cellular component ontology), 684 (molecular function ontology) and 538 (biological process ontology) genes were identified between the mock and late infection samples. Furthermore, specific enrichment of genes was observed in GO terms including insoluble fraction, peptidase activity and cellular amino acid metabolic process in the late infection sample. KEGG annotation revealed that 1232 differentially expressed genes were identified in the late infection samples. Notably, enrichment of genes was observed for pathways including ECM-receptor interaction, complement and coagulation cascades, antigen processing and presentation etc.

Furthermore, the differentially expressed genes were analyzed among three samples. Many genes in early infection samples (more than 44.1% of the up-regulated genes and 67.0% of the down regulated genes) and late infection samples (56.8% of the up-regulated genes and 65.8% of the down-regulated genes) are orphan sequences-no homologues found in the NCBI database.

### Experimental Validation of Transcriptome and DGE Analysis

Eight unigenes were chosen from sequencing and computational analysis to confirm the characterizations of expression by using RT-PCR and qRT-PCR analysis. The primers used in the validation experiments were shown in Table 4.

**Table 4:** Primers used in the validation experiment.

Unigene number	Similarity	RT-PCR and qRT-PCR
		Primers Sequences
unigene18988	chymotrypsin BI	GCCAGCCAGGTCTCCATT
		GGAGTGACGCCGGTCTTCT
unigene8925	ubiquitin-conjugating enzyme E2	CAACGAATCAGCAAGGAG
		CTGGCGGGTCCACTCAC
unigene7508	argonaute 2	TGTAAGGCTCAAACTGGA
		TGGAGCAGCTCTTATCAC
unigene1876	heat shock protein 70	TCTCGGGTCTGAATGTGC
		ACGGGTGATGGAGGTGTA
unigene18451	penaeidin4a	AAGGCGAAGCGTACAGGG
		CGAGCATCTGAGACGGAAA
unigene18880	peroxinectin	ATGCTTTCTATGGACCTCG
		CCACAAACCTTCTAGCCTCT
unigene9038	glucan binding protein	GTTGACTGGACCAAGGAGA
		GAAGAAGCCGTTCGTGCC
unigene9387	peroxiredoxin	AGCCTTCAAGTTCACA
		ATCTAATGCCATATCCT

**Figure 8:**
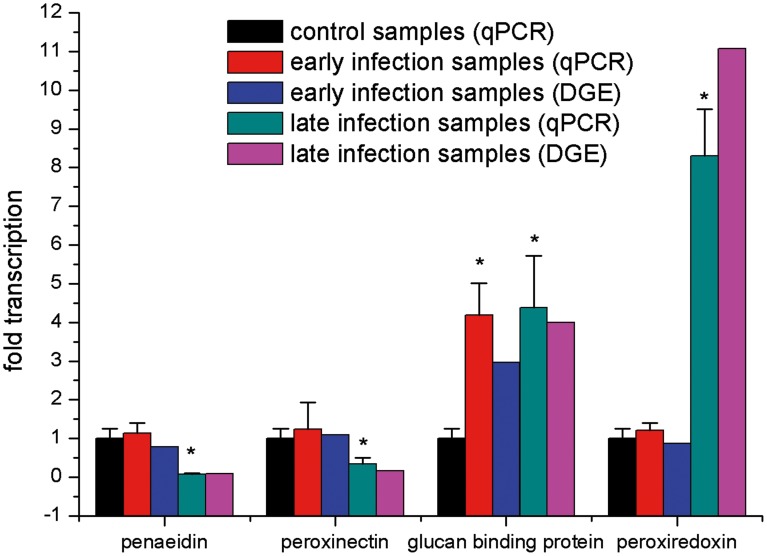
Real-time PCR analysis of four selected genes from *L. vannamei* which showed different expression after WSSV infection based on deep sequencing analysis. Bars with asterisks indicated values that were significantly different (P<0.05) from control samples. Error bars indicated standard deviations of averages from three replicates.

In RT-PCR analysis, all the examined genes matched the RNA-seq-generated sequences perfectly (data not shown). It shows the reliability of RNA-seq results and indicates the necessity for further identification of immune-related genes in *L. vannamei.* In qRT-PCR analysis, no significant differences of the expression levels of penaeidin, peroxiredoxin and peroxinectin were observed between early infection samples and control samples except that glucan-binding protein was highly expressed (P<0.05) in early infection samples. In the late infection samples, penaeidin and peroxinectin were down-regulated significantly (P<0.05), while glucan-binding protein and peroxiredoxin were up-regulated significantly (P<0.05). The results of expression profiles by qRT-PCR analysis had the same variation trend with the DGE data (Figure 8).

## Discussion

Transcriptome is essential in deciphering the functional complexity of cellular activities including diseases, immune defense, growth and development. Traditionally, gene expression analysis of shrimp has relied mostly on expressed sequence tag (EST) [30]–[34], serial analysis of gene expression (SAGE) [35], and cDNA microarrays [36]–[37]. However, each of those approaches has some inherent limitations such as cloning biases, costly and need prior sequence knowledge and so on. Thus, the newly developed deep sequencing approaches have significant changes on how to investigate the functional complexity of the transcriptome.

In the present study, the Illumina sequencing method was used to analyze the transcriptome of *L. vannamei* challenged with WSSV. 52,073 unigenes were assembled by 25.81 million clean reads. The large scale data will provide a good foundation for further research into the new gene discovery, genetics, genomics and even proteomics of shrimp.

In addition, differentially expressed genes in the early and late WSSV infection stage were analyzed by using DGE method which generates absolute rather than relative gene expression measurements. A total of 6.25, 6.02 and 5.98 million high quality non-redundant tags of the mock, early and late infection samples were identified, respectively. Through assessment of saturation and distribution of DGE tags, it is suggested that the data was good enough for further gene expression analysis.

DGE mapping analysis revealed that only 315 differentially expressed genes were identified, including 227 up-regulated and 88 down-regulated genes, with 100 and 59 orphan sequences-no homologues found in the NCBI database, respectively. As we know, WSSV can trigger a series of immediate-early (IE) and early genes to encode proteins involved in activating the expression of viral early and late genes, altering the functions of host genes and eliminating host immune defense [38]–[45]. It was speculated that the strategies which WSSV deployed are more instantaneous and accurate than the immune defense of shrimp. Even though, the expression of some classical immune-related genes including pattern recognition proteins (C-type lectin, glucan-binding protein), hemocyanin and prophenoloxidase showed most up-regulated in the early WSSV infection stage. The recognition of microorganisms is the first step in the immune process, and then a whole series of mechanisms, such as phagocytosis, encapsulation and nodule formation, is triggered. Hemocyanin is an important innate immune response against WSSV, since it is capable to delay WSSV infection [46]. Prophenoloxidase activation system (proPO-AS) can trigger a serine proteinase cascade and plays an important role in the invertebrate immune system. The highly expression of prophenoloxidase showed that the proPO-AS system was activated by WSSV. Furthermore, it was also found that all the down-regulated genes annotated in the NCBI database had relative slightly change with decreasing less than 4 fold in this research. From the above results, it is indicated that WSSV could trigger some immune responses of shrimp, but the response was relatively mild.

In the late phase of WSSV infection, a total of 1,672 genes which was annotated in the NCBI database were identified, with 1,083 up-regulated and 589 ones down-regulated. The transcriptional level of abundant innate immune-related genes, including pattern recognition proteins, phenoloxidase system revelent, antioxidant enzymes, programmed cell death protein, ubiquitin proteasome pathway revelent, cell adhesion molecules, signal transduction molecule and other immune response factors, were changed. It indicated that the shrimp immune system was fully activated by the virus. It is interesting that a large number of differentially expressed genes involved in glycolytic pathway, oxidative phosphorylation, cytoskeleton, calicium metabolism, amino acid metabolism, nucleotide metabolism and even nerve system, were also identified which might imply that the metabolic balance of shrimp is disorder under WSSV infection. It also could give us some clues to explain the symptoms such as white spot, loose cuticle, and hypoxia appeared on the shrimp in the late infection phase. In the game of virus and shrimp, WSSV is usually the winner upon most occasions since high mortality of shrimp infected by the virus. It could be concluded that some of the differentially expressed genes may not only participate in the immune defense of shrimp, but are recruited by the virus for its successful infection. Recently, studies showed that silencing of some immune-related genes had delayed the mortalities of shrimp and decreased the copies of WSSV [16], [47]. To understand the precise roles these differentially expressed genes, further studies of function and regulation on them are in progress.

## Conclusions

In conclusion, the 52,073 high-quality unigenes and DGE data generated in the present study provide a rich source for identification of novel genes in shrimp and for comparative analysis of gene expression patterns in normal and WSSV-infected shrimp. Our data suggested that the shrimp immune system was slight changed in the early WSSV infection phase. However, WSSV infection could strongly activate the immune responses of shrimp and made the metabolism balance disorder in the late infection stage.

## Supporting Information

Table S1 Details on immune-related genes/homologues in *L. vannamei*.
(XLSX)

Table S2 Differentially expressed genes between the mock and early infection samples.
(XLS)

Table S3 Differentially expressed genes between the mock and late infection samples.
(XLS)

Table S4 Immune-related genes screened from the late infection samples.
(XLS)
